# Vaccination program in a resource-limited setting: A case study in the Philippines

**DOI:** 10.1016/j.vaccine.2016.08.014

**Published:** 2016-09-14

**Authors:** Sarocha Chootipongchaivat, Varit Chantarastapornchit, Wantanee Kulpeng, Joyce Anne Ceria, Niña Isabelle Tolentino, Yot Teerawattananon

**Affiliations:** a Health Intervention and Technology Assessment Program (HITAP), 6th Floor, 6th Building, Department of Health, Ministry of Public Health, Tiwanon Rd., Mueng, Nonthaburi 11000, Thailand; b Pharmaceutical Division (PD) - Office for Health Regulation (OHR), 3rd Floor, Bldg. 15, Department of Health, San Lazaro Compound, Rizal Avenue, Sta Cruz, 1003 Manila, Philippines

**Keywords:** Pneumococcal conjugate vaccine, Human resources for health, HRH, PCV, Philippines, Vaccination coverage

## Abstract

**Objective:**

Implementing national-level vaccination programs involves long-term investment, which can be a significant financial burden, particularly in resource-limited settings. Although many studies have assessed the economic impacts of providing vaccinations, evidence on the positive and negative implications of human resources for health (HRH) is still lacking. Therefore, this study aims to estimate the HRH impact of introducing pneumococcal conjugate vaccine (PCV) using a model-based economic evaluation.

**Methods:**

This study adapted a Markov model from a prior study that was conducted in the Philippines for assessing the cost-effectiveness of 10-valent and 13-valent PCV compared to no vaccination. The Markov model was used for estimating the number of cases of pneumococcal-related diseases, categorized by policy options. HRH-related parameters were obtained from document reviews and interviews using the quantity, task, and productivity model (QTP model).

**Results:**

The number of full-time equivalent (FTE) of general practitioners, nurses, and midwives increases significantly if the universal vaccine coverage policy is implemented. A universal coverage of PCV13 - which is considered to be the best value for money compared to other vaccination strategies - requires an additional 380 FTEs for general practitioners, 602 FTEs for nurses, and 205 FTEs for midwives; it can reduce the number of FTEs for medical social workers, paediatricians, infectious disease specialists, neurologists, anaesthesiologists, radiologists, ultrasonologists, medical technologists, radiologic technologists, and pharmacists by 7, 17.9, 9.7, 0.4, 0.1, 0.7, 0.1, 12.3, 2, and 9.7, respectively, when compared to the no vaccination policy.

**Conclusion:**

This is the first attempt to estimate the impact of HRH alongside a model-based economic evaluation study, which can be eventually applied to other vaccine studies, especially those which inform resource allocation in developing settings where not only financial resources but also HRH are constrained.

## 1 Introduction

Human resources are an integral part of every healthcare system [1]. However, in low- and middle-income countries (LMICs), human resources for health (HRH) are often limited, which impacts both the access to and quality of healthcare [1], [2]. The shortage of healthcare workers can be due to different factors such as low production capacity for HRH, brain drain of healthcare workers, inefficient use of human resources or imbalance in the composition of demographics [3], [4]. As the demand for healthcare services increases, this scarcity can result in instability of the healthcare system. In addition, introducing health interventions or technologies will always have an effect on the demand for human resources and this should be a serious concern for decision-makers, especially in LMICs, as training health workforce (i.e. general practitioners and medical specialists) requires a considerable amount of time [5], [6], [7].

Vaccination can be considered as a unique intervention in the context of human resources. Vaccination programs are a long-term investment for preventing specific diseases, and have a dynamic effect on the utilization of different types of HRH. Vaccination programs could increase the present need of particular types of human resources and reduce the future need of the health workforce in treating vaccine preventable diseases [8], [9]. Although there are few papers addressing the success of vaccination in terms of HRH requirements [2], [10], no study has been conducted that compares the impact of vaccinations in terms of both HRH needed and reduced within a study.

It would be appropriate for evidence-informed policy decisions to take into account both HRH required and saved due to vaccinations. Therefore, this study aims to take this challenge by estimating the HRH needed and reduced as a result of introducing the pneumococcal conjugate vaccine (PCV). This study is conducted as an additional analysis to the economic evaluation using the Philippines as a case study due to their existing economic model which compares various vaccination policy options [11].

## 2 Material and methods

This study examines the impact of HRH by using the quantity, task, and productivity (QTP) model. The QTP model is one of the approaches to determine HRH and was developed under the concept of functional job analysis whereby the skill requirements to complete a certain task are assessed [12]. There are four main key features of this model: (1) it includes a set of priority interventions, (2) it estimates HRH by calculating the number of cases needed for a service (service quantity), (3) it identifies the tasks and estimates the time needed to deliver a service, and (4) it includes the productivity by combining staff productivity and service productivity. This model has been developed for low-income countries that want to scale up their priority interventions. A study conducted by Kurowski et al. showed that the QTP method was robust in estimating the required human resources [13].

**Fig. 1:**
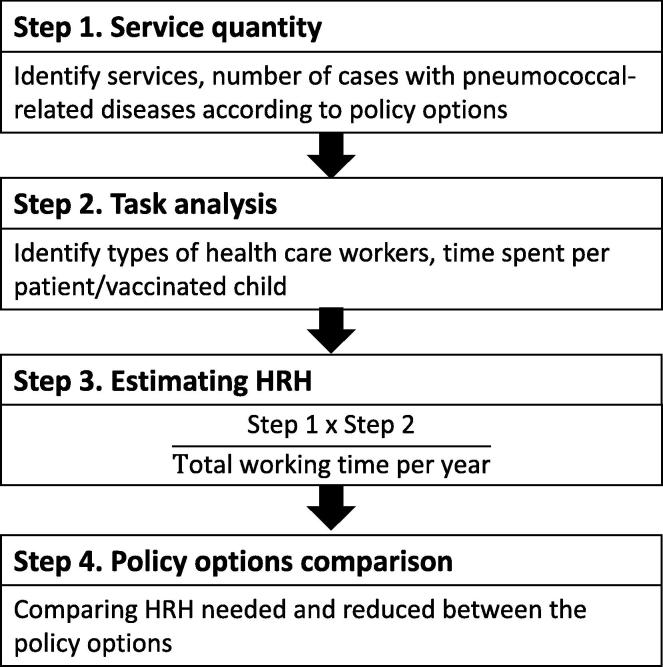
Summary of the four steps in HRH estimation.

Furthermore, this model is practical and feasible for application in the Philippines where data resources are restricted. The adapted version of the QTP model for estimating the HRH impact of introducing the PCV vaccination in the Philippines is shown in Supplement A. Using this model, the HRH-related parameters were obtained from existing clinical practice guidelines [14], [15], [16], [17] wherein the applicability of each procedure in the local setting was verified by conducting interviews with healthcare providers. The adapted QTP model includes four major steps (Fig. 1) and is described in the following paragraphs.

In the first step, several health services for the treatment of pneumococcal-related diseases were identified, including the number of cases that occurred in each scenario, i.e. with and without the vaccination program. The number of cases for each disease was estimated using the Markov model from a prior economic evaluation study that was conducted in the Philippines [11]. One-year time horizon was employed in this study, while the prior economic evaluation used a Markov model with a lifetime horizon. The target population for universal coverage of the PCV vaccination was set at 2,200,000 eligible infants below the age of one year based on 2013 data obtained from the Philippine Statistics Authority [18].

In the second step, the types of health workforces needed for each type of health service relating to vaccination, treatments of non-hospitalized pneumonia, and acute otitis media were obtained based on consensus among municipal health officers in the seminar for the primary care benefit package of the state insurance scheme; meanwhile, data on treatment of meningitis, sepsis/bacteraemia and hospitalized pneumonia in intensive care units (ICU) and non-ICUs were derived from four medical specialists. In the data collection process, participants were given information on healthcare services as stated by the clinical practice guidelines from the Philippine Health Insurance Corporation [14]; Philippine Clinical Practice Guidelines on the Diagnosis, Empiric Management, and Prevention of Community-acquired Pneumonia (CAP) in Immunocompetent Adults [15]; and Integrated Management of Childhood Illness (IMCI) [16], [17]. After that, they were asked to indicate the set of healthcare services they provide in their practice, followed by identifying the types of health workforce needed as well as the average amount of time (minutes) for each health professional spent per treated patient or vaccinated child.

In the third step, the magnitude of HRH needed for each policy option was estimated. Staff productivity was assumed to be 6 h or 360 min per day, with 220 working days in a year. The number of healthcare workers needed for the implementation of the PCV vaccination and for the treatment of pneumococcal-related diseases was calculated in full-time equivalent (FTE) using the formula below. One FTE equals one employee that works on a full-time basis. In the last step, HRH needed and reduced were compared with each other for each policy option.

$$\text{Number of healthcare workers}\;\;(\text{in FTEs})=\frac{\text{Total time spent of healthcare provider per case}\;(\text{minutes})\times \text{Number of cases or target population}}{\text{Total working time per year}\;(=360*220\;\text{min}/\text{person}/\text{year})}$$

## 3 Results

Based on five available policy options (no vaccination program, PCV10 with 25 percent coverage, PCV10 with 100 percent coverage, PCV13 with 25 percent coverage, and PCV13 with 100 percentage coverage), Table 1 illustrates six types of healthcare services that are related to the prevention or treatment of pneumococcal-related diseases: vaccination, meningitis treatment, sepsis/bacteraemia treatment, hospitalized pneumonia treatment, non-hospitalized pneumonia treatment, and acute otitis media treatment. Regardless of the vaccination program, the demand for acute otitis media treatment is the highest, followed by the pneumonia treatment. Implementing the PCV10 or PCV13 would reduce the number of the aforementioned vaccine-preventable conditions. The higher the vaccination coverage, the lower the number of patients treated.

Table 2 presents the average amount of time that each type of health professional spends per treated patient or vaccinated child. The average time spent per case is the highest for the treatment of meningitis by paediatricians, which equals 746.50 min per case. The lowest average time spent per care is for midwives, which is only 5 min for non-hospitalized pneumonia and acute otitis media treatment, and for radiologists, which is 5 min for both hospitalized and non-hospitalized pneumonia treatment.

**Fig. 2:**
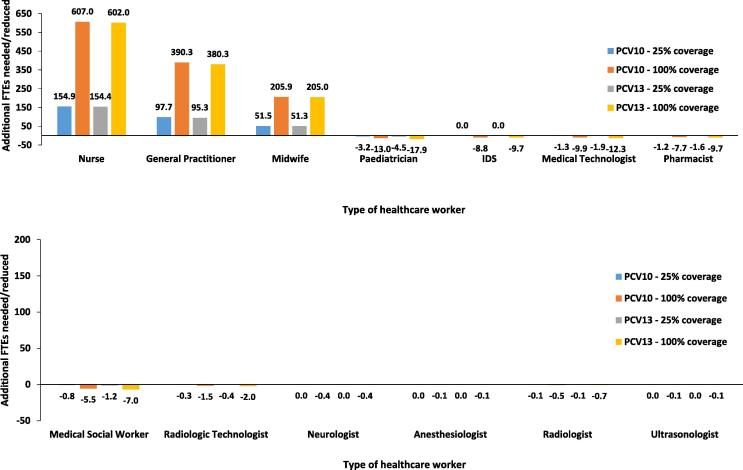
Additional HRH needed and reduced.

Further, the number of healthcare professionals needed for each policy option was identified in Table 3. This table and Fig. 2 show the increase and reduction of FTEs for each type of healthcare professionals required for the treatment of pneumococcal-related diseases resulting from the implementation of the PCV vaccination policy. It can be seen that the implementation of the PCV vaccination significantly increases the number of general practitioners, nurses, and midwives required for the vaccination program.

## 4 Discussion

This is the first attempt to estimate the impact of HRH alongside a model-based economic evaluation study, which can be eventually applied to other studies, especially those that inform resource allocation in developing settings where not only financial resources but HRH are also constrained. This study is different from economic evaluations, which focus only on comparisons between costs and outcomes (Incremental Cost-Effectiveness Ratio; ICER). Although economic evaluation guidelines recommend reporting costs and resources used separately [19], [20], only few papers do this [21], [22] and even fewer papers report resources used by giving detailed information of health workforce. This HRH study is a complementary analysis and the results are reported as HRH saved and needed (in terms of FTE). The results showed that the number of FTEs for GPs, nurses, and midwives increases significantly if the universal vaccination coverage policy is implemented. Nevertheless, the vaccination program can avert HRH requirement for specialized healthcare professionals who require longer term training and are more restricted compared to GPs, nurses, and midwives in the Philippines [23]. Moreover, the salary of GPs, nurses, and midwives are likely to be lower than those specialized healthcare professionals.

An associated cost-effectiveness analysis found that the universal coverage for PCV13 has the lowest ICER compared to no vaccination amongst the four vaccination strategies [11]. Although this policy needs an additional 380 FTEs for general practitioners, 602 FTEs for nurses, and 205 FTEs for midwives, it can reduce the number of FTEs for medical social workers, paediatricians, infectious disease specialists, neurologists, anaesthesiologists, radiologists, ultrasonologists, medical technologists, radiologic technologists and pharmacists by 7, 17.9, 9.7, 0.4, 0.1, 0.7, 0.1, 12.3, 2, and 9.7, respectively, when compared to the no vaccination policy. From the analysis can also be observed that HRH requirements for both PCV10 and PCV13 were very similar, meaning that choosing PCV13 over PCV10 would not have differed in terms of HRH impact. This type of information should be presented to decision-makers so that they can make an appropriate and feasible policy choice, taking into account not only cost-effectiveness evidence and budget impact but also impact on HRH, which cannot be increased within a short period of time. Neglecting information on HRH when introducing any large health programs, including vaccination, can put the policy at high risk of failure due to the overburden of existing healthcare workers.

If the estimated HRH required seems infeasible for implementing a universal vaccination program, a proper plan for task shifting of vaccination activities – i.e. physical examinations that are being carried out by GPs - to nurses, midwives, and pharmacists should be investigated. Meanwhile, FTEs of specialized healthcare professionals freed up by the vaccination program can be used for other health policies. In addition, it is interesting to note that only a relatively small reduction of FTEs among specialized healthcare workers from the vaccination program was observed in this study. An explanation could be that in the Philippines, there is a severely limited number of these specialists and they are working under high workloads, resulting in limited time spent for each patient treated. The prevention of pneumococcal-related diseases from the vaccination means that they can spend a longer time providing better care for patients. Although this study did not investigate whether the current time allocated to the treatment of pneumococcal-related diseases was appropriate, the current estimates indicate that the quality of care may be less than optimal due to shortages of key staff.

This study has some limitations. Firstly, the profile (type) and magnitude (time spent) of health professionals required for each service are specific to the context of the Philippines; therefore, the use of this study’s results for other settings needs to be performed with caution. Secondly, the study focuses on the impact of HRH at the national level. However, the aforementioned HRH may not be distributed equally across geographical locations, especially between urban and rural areas [24]. It would be necessary to estimate the HRH impact for each province when introducing the vaccination policy in order to ensure that HRH planning is adequate across sub-national levels. Thirdly, this study only considered a one-year time horizon despite the fact that previous evidence suggested that the vaccine may be able to prevent pneumococcal-related diseases for up to 5 years after vaccination [25], which may result in an underestimation of HRH freed up by the vaccine program. However, this study already included herd protection for universal vaccination coverage but not for 25 percent coverage. Lastly, this study employed an expert opinion approach for data collection on the number of minutes used by health professionals in treating each child and this can be problematic in terms of robustness. Although experts were requested to discuss about the estimates among their peers in order to ensure that the provided numbers were feasible, this might not be an ideal approach. With this regard, it is recommended for future study to conduct a prospective observational data collection. The benefit of collecting primary data from observation is that a probabilistic sensitivity analysis can be performed to assess the uncertainty on parameters.

There are two interesting points for further study: 1) to explore the effects of adherence to clinical practice guidelines for physicians on estimating HRH in comparison to real practice, and 2) to consider whether there is a need for discounting if the HRH impact is estimated beyond a one-year time horizon. For example, when implementing the human papilloma virus (HPV) vaccination program at present, the effects on HRH (e.g., cervical oncologists) will occur in the next twenty or thirty years. In such case, it is questionable whether the effects on HRH in the future should be discounted similarly to the standard practice in economic evaluations that discounts further costs and outcomes [19]. Furthermore, the future impact of technology and innovation will have unknown implications on the HRH required in terms of health management. Any adjustments for predicted HRH in the future may introduce considerable uncertainty and may limit future HRH planning.

## 5 Conclusions

This study examines an approach for estimating HRH impact alongside economic evaluation studies on PCV vaccination policy in the Philippines. It illustrates the importance of HRH impact estimations to inform policy decisions in resource-limited settings; this is to ensure that comprehensive evidence was used to formulate policy choice for decision makers. The study informs the reduction of specialized healthcare professionals for treatment of pneumococcal-related diseases vis-a-vis the increase of HRH requirement among GPs, nurses, and midwives as a result of implementing a PCV vaccination program. The authors conclude that the HRH impact should be estimated to inform priority setting for a vaccination program.

## Conflict of interest statement

None.

## Figures and Tables

**Table 1:** Number of cases receiving health care services by policy options.

Types of health care services	Policy options				
	1	2	3	4	5
	No vaccination program	PCV10 with 25% coverage of vaccination program	PCV10 with 100% coverage of vaccination program	PCV13 with 25% coverage of vaccination program	PCV13 with 100% coverage of vaccination program
Vaccination	0	550,000	2,200,000	550,000	2,200,000
Meningitis treatment	3331	3305	2807	3295	2747
Sepsis/ Bacteremia treatment	3260	3247	2679	3242	2612
Hospitalized pneumonia treatment	327,714	326,947	323,592	326,654	322,272
Non-hospitalized pneumonia treatment	218,476	217,965	215,728	217,769	214,848
Acute otitis media treatment	2,550,431	2,541,432	2,514,437	2,537,981	2,500,631

**Table 2:** Average amount of time that each type of health professional spent per vaccinated child/adult⁎.

Types of health care services	Average time spent per case (min)												
	Medical Social Worker	General Practitioner	Pediatrician	IDS (adult)	Neurologist	Anesthesiologist	Radiologist	Ultrasonologist	Medical Technologist	Radiologic Technologist	Pharmacist	Nurse	Midwife
Vaccination	-	15	-	-	-	-	-	-	-	-	-	22.5	7.5
Meningitis treatment	82.5	-	746.5	562.5	58.8	15	-	13.5	117.5	-	315	630	-
								17.5^∗^	345^∗^		195^∗^	390^∗^	

Sepsis/ bacteremia treatment	90	-	529	404	-	-	10	-	165	8	210	420	-
									128.8^∗^		150^∗^	300^∗^	

Hospitalized pneumonia treatment	82.5	-	300.3	237.8	-	-	5	-	117.5	17.5	105	210	-
										12.8^∗^	75^∗^	150^∗^	

Non-hospitalized pneumonia treatment	-	72.5	-	-	-	-	5	-	22.5	17.5	-	37.5	5

Acute otitis media treatment	-	52.5	-	-	-	-	-	-	-	-	-	7.5	5

⁎ Treatment time of an adult.

**Table 3:** Number of full time equivalent for each type of health care worker for each policy option.

Types of health care worker	Policy options				
	1	2	3	4	5
	No vaccination program	PCV10 with 25% coverage of vaccination program	PCV10 with 100% coverage of vaccination program	PCV13 with 25% coverage of vaccination program	PCV13 with 100% coverage of vaccination program
Medical Social Worker	348.5	347.4	341.5	347.7	343.0
General Practitioner	1890.6	1985.9	2271.0	1988.4	2280.9
Pediatrician	961.1	956.6	943.2	957.9	948.2
IDS	263.9	263.9	254.2	263.9	255.0
Neurologist	2.5	2.4	2.0	2.5	2.1
Anesthesiologist	0.6	0.6	0.5	0.6	0.5
Radiologist	34.9	34.8	34.2	34.8	34.4
Ultrasonologist	0.6	0.6	0.5	0.6	0.5
Medical Technologist	563.3	561.5	551.0	562.0	553.5
Radiologic Technologist	116.2	115.8	114.2	115.9	114.7
Pharmacist	421.9	420.3	412.2	420.7	414.2
Nurse	1185.9	1340.3	1787.9	1341.6	1793.7
Midwife	174.8	226.1	379.8	226.3	380.7

## References

[1] Hongoro C., McPake B. (2004). How to bridge the gap in human resources for health. Lancet.

[2] Chen L., Evans T., Anand S., Boufford J.I., Brown H., Chowdhury M. (2004). Human resources for health: overcoming the crisis. Lancet.

[3] Kollar E., Buyx A. (2013). Ethics and policy of medical brain drain: a review. Swiss Med Wkly.

[4] Kinfu Y., Dal Poz M.R., Mercer H., Evans D.B. (2009). The health worker shortage in Africa: are enough physicians and nurses being trained?. Bull World Health Organ.

[5] Hauck K., Thomas R., Smith P.C. (2016). Departures from cost-effectiveness recommendations: the impact of health system constraints on priority setting. Health Syst Reform.

[6] Biellik R., Levin C., Mugisha E., LaMontagne D.S., Bingham A., Kaipilyawar S. (2009). Health systems and immunization financing for human papillomavirus vaccine introduction in low-resource settings. Vaccine.

[7] Jha V., Garcia-Garcia G., Iseki K., Li Z., Naicker S., Plattner B. (2013). Chronic kidney disease: global dimension and perspectives. Lancet.

[8] Bärnighausen T., Bloom D.E., Cafiero E.T., O’Brien J.C. (2013). Valuing the broader benefits of dengue vaccination, with a preliminary application to Brazil. Seminars in immunology.

[9] Shepard D.S., Suaya J.A., Halstead S.B., Nathan M.B., Gubler D.J., Mahoney R.T. (2004). Cost-effectiveness of a pediatric dengue vaccine. Vaccine.

[10] Anand S., Bärnighausen T. (2007). Health workers and vaccination coverage in developing countries: an econometric analysis. Lancet.

[11] Haasis M.A., Ceria J.A., Kulpeng W., Teerawattananon Y., Alejandria M. (2015). Do pneumococcal conjugate vaccines represent good value for money in a lower-middle income country? A cost-utility analysis in the Philippines. PLoS ONE.

[12] Kurowski C., Mills A. (2006). Estimating human resource requirements for scaling up priority health interventions in low income countries of Sub-Saharan Africa: a methodology based on service quantity, tasks and productivity.

[13] Kurowski C., Wyss K., Abdulla S., Mills A. (2007). Scaling up priority health interventions in Tanzania: the human resources challenge. Health Policy Plann.

[14] PhilHealth (2006). Performance report on PhilHealth use of CPGs for quality assurance and accreditation. HTA Forum.

[15] Joint statement of the Philippine Society for Microbiology and Infectious Diseases PCoCP, Philippine Academy of Family Physicians, Philippine College of Radiology. Philippine clinical practice guidelines on the diagnosis, empiric management, and prevention of community-acquired pneumonia (CAP) in immunocompetent adults; 2010. <http://www.psmid.org.ph/clinical/cap_guidelines_2010.pdf>.

[16] World Health Organization. Midwifery education modules; 2008. <http://apps.who.int/iris/bitstream/10665/44145/6/9789241546669_6_eng.pdf>.

[17] World Health Organization (2003). Managing newborn problems: a guide for doctors, nurses, and midwives. http://apps.who.int/iris/bitstream/10665/42753/1/9241546220.pdf.

[18] Philippine Statistics Authority. Projected populations by five-year age group and sex, by region and province, and by five-calendar years: 2000–2040, 2006.

[19] Canadian Agency for Drugs Technologies in Health (2006). Guidelines for the economic evaluation of health technologies.

[20] World Health Organization (2008). WHO guide for standardization of economic evaluations of immunization programmes.

[21] Bhattarai N., McMeekin P., Price C., Vale L. (2016). Economic evaluations on centralisation of specialised healthcare services: a systematic review of methods. BMJ Open.

[22] Vijgen S., Opmeer B.C., Mol B. (2013). The methodological quality of economic evaluation studies in obstetrics and gynecology: a systematic review. Am J Perinatol.

[23] Romualdez A.G., dela Rosa J.F., Fla vier J.D.A., Quimbo S.L.A., Hartigan-Go K.Y., Lagrada L.P. (2011). The Philippines health system review.

[24] Finch S. (2013). Philippines brain drain: fact or fiction?. Can Med Assoc J.

[25] Madhi S.A., Adrian P., Kuwanda L., Jassat W., Jones S., Little T. (2007). Long-term immunogenicity and efficacy of a 9-valent conjugate pneumococcal vaccine in human immunodeficient virus infected and non-infected children in the absence of a booster dose of vaccine. Vaccine.

