# Supplementary material for: Vaccination program in a resource-limited setting: A case study in the Philippines
Source: Vaccine. 2016 Sep 14;34(40):4814–9. doi: 10.1016/j.vaccine.2016.08.014 (PMC5022401; doi:10.1016/j.vaccine.2016.08.014)
Supplement: Supplementary data 1 [file mmc1.docx]

**Supplement A. The quantity, task and productivity (QTP) model**

| Implementing intervention(s) | | | | | | | | | | | | | | |
| --- | --- | --- | --- | --- | --- | --- | --- | --- | --- | --- | --- | --- | --- | --- |
| Step 1: Estimating the quantity of needed services | | | | | | | | | | | | | | |
| Number of population related to diseases or health conditions | | | | | | | |  | | | | | | |
| Incidence of diseases or health conditions related to intervention | | | | | | | |  | | | | | | |
| Expecting number of target population | | | | | | | |  | | | | | | |
| Step 2: Task analysis | | | | | | | | | | | | | | |
|  | Time weight | Skill level | | | | | | | | | | Infrastructure level | | |
| First visit/ visit for a specific purpose e.g. lab test | (Mins) | HRH1 | | HRH2 | | HRH3 | | | HRH4 | | | A | B | C |
| Task 1 |  |  | |  | |  | | |  | | |  |  |  |
| Task 2 |  |  | |  | |  | | |  | | |  |  |  |
| Task 3 |  |  | |  | |  | | |  | | |  |  |  |
| Task 4 |  |  | |  | |  | | |  | | |  |  |  |
| Task 5 |  |  | |  | |  | | |  | | |  |  |  |
| Second visit/ visit for a specific purpose e.g. lab test | (Mins) | HRH1 | | HRH2 | | HRH3 | | | HRH4 | | | A | B | C |
| Task 1 |  |  | |  | |  | | |  | | |  |  |  |
| Task 2 |  |  | |  | |  | | |  | | |  |  |  |
| Task 3 |  |  | |  | |  | | |  | | |  |  |  |
| Task 4 |  |  | |  | |  | | |  | | |  |  |  |
| Task 5 |  |  | |  | |  | | |  | | |  |  |  |
| Third visit / visit for a specific purpose e.g. lab test | (Mins) | HRH1 | | HRH2 | | HRH3 | | | HRH4 | | | A | B | C |
| Task 1 |  |  | |  | |  | | |  | | |  |  |  |
| Task 2 |  |  | |  | |  | | |  | | |  |  |  |
| Task 3 |  |  | |  | |  | | |  | | |  |  |  |
| Task 4 |  |  | |  | |  | | |  | | |  |  |  |
| Task 5 |  |  | |  | |  | | |  | | |  |  |  |
| The time weights across all contacts of the intervention can be summarized by skill level and infrastructure level as follows: | | | | | | | | | | | | | | |
| Skill level | A | | | | B | | | | | C | | | | |
| 1 |  | | | |  | | | | |  | | | | |
| 2 |  | | | |  | | | | |  | | | | |
| 3 |  | | | |  | | | | |  | | | | |
| 4 |  | | | |  | | | | |  | | | | |
| Step 3: Estimating HRH requirements as FTE’s | | | | | | | | | | | | | | |
| Net work days per annual | | | | | | | |  | | | | | | |
| Working hours per day of full time employees | | | | | | | |  | | | | | | |
| [FTE] is the full-time equivalent calculated = [net work days per year]*[working hours per day]*60 | | | | | | | |  | | | | | | |
| HRH requirement for meningitis  = [Number of meningitis cases]*[task matrix] / [FTE] | | | | | | | | | | | | | | |
| Skill level | A | | | | B | | | | | C | | | | |
| 1 |  | | | |  | | | | |  | | | | |
| 2 |  | | | |  | | | | |  | | | | |
| 3 |  | | | |  | | | | |  | | | | |
| 4 |  | | | |  | | | | |  | | | | |
| Step 4: Comparing the different HRH between policy options | | | | | | | | | | | | | | |
| Policy options | HRH1 | | HRH2 | | | | HRH3 | | | | HRH4 | | | |
| 1 |  | |  | | | |  | | | |  | | | |
| 2 |  | |  | | | |  | | | |  | | | |
| 3 |  | |  | | | |  | | | |  | | | |
| 4 |  | |  | | | |  | | | |  | | | |
